# Evolution of gastropod mitochondrial genome arrangements

**DOI:** 10.1186/1471-2148-8-61

**Published:** 2008-02-26

**Authors:** Cristina Grande, José Templado, Rafael Zardoya

**Affiliations:** 1 Departamento de Biodiversidad y Biología Evolutiva, Museo Nacional de Ciencias Naturales, CSIC, José Gutiérrez Abascal, 2, 28006, Madrid, Spain; 2 Department of Integrative Biology, University of California, Berkeley, CA, USA

## Abstract

**Background:**

Gastropod mitochondrial genomes exhibit an unusually great variety of gene orders compared to other metazoan mitochondrial genome such as e.g those of vertebrates. Hence, gastropod mitochondrial genomes constitute a good model system to study patterns, rates, and mechanisms of mitochondrial genome rearrangement. However, this kind of evolutionary comparative analysis requires a robust phylogenetic framework of the group under study, which has been elusive so far for gastropods in spite of the efforts carried out during the last two decades. Here, we report the complete nucleotide sequence of five mitochondrial genomes of gastropods (*Pyramidella dolabrata*, *Ascobulla fragilis*, *Siphonaria pectinata*, *Onchidella celtica*, and *Myosotella myosotis*), and we analyze them together with another ten complete mitochondrial genomes of gastropods currently available in molecular databases in order to reconstruct the phylogenetic relationships among the main lineages of gastropods.

**Results:**

Comparative analyses with other mollusk mitochondrial genomes allowed us to describe molecular features and general trends in the evolution of mitochondrial genome organization in gastropods. Phylogenetic reconstruction with commonly used methods of phylogenetic inference (ME, MP, ML, BI) arrived at a single topology, which was used to reconstruct the evolution of mitochondrial gene rearrangements in the group.

**Conclusion:**

Four main lineages were identified within gastropods: Caenogastropoda, Vetigastropoda, Patellogastropoda, and Heterobranchia. Caenogastropoda and Vetigastropoda are sister taxa, as well as, Patellogastropoda and Heterobranchia. This result rejects the validity of the derived clade Apogastropoda (Caenogastropoda + Heterobranchia). The position of Patellogastropoda remains unclear likely due to long-branch attraction biases. Within Heterobranchia, the most heterogeneous group of gastropods, neither Euthyneura (because of the inclusion of *P. dolabrata*) nor Pulmonata (polyphyletic) nor Opisthobranchia (because of the inclusion *S. pectinata*) were recovered as monophyletic groups. The gene order of the Vetigastropoda might represent the ancestral mitochondrial gene order for Gastropoda and we propose that at least three major rearrangements have taken place in the evolution of gastropods: one in the ancestor of Caenogastropoda, another in the ancestor of Patellogastropoda, and one more in the ancestor of Heterobranchia.

## Background

The animal mitochondrial (mt) genome is a circular double-stranded DNA molecule, which typically encodes for two rRNAs, 22 tRNAs, and 13 proteins that are essential for mitochondrial function [1]. The advent of long PCR and automated sequence techniques has recently simplified and accelerated the determination of complete sequences (about 16 Kb) of animal mtDNAs [2], and as of May 2007 there were more than 1000 complete metazoan mtDNAs deposited in GenBank [3]. Most (73%) of these sequences were from vertebrate mtDNAs, which show a relatively fixed genome organization with only few instances of gene rearrangements [4,5]. Instead, changes in gene order are widespread in non-vertebrate mt genomes, and particularly frequent in several phyla such as e.g. nematodes or mollusks [6]. Tandem duplication followed by random loss of redundant genes has been demonstrated to be the main mechanism for gene rearrangement of adjacent tRNAs in vertebrate mt genomes [5]. However, other mechanisms such as illegitimate recombination mediated by e.g. topoisomerases need to be invoked to explain observed gene inversions, as well as transpositions of genes to distant positions in non-vertebrate mt genomes [6]. In order to determine rates and mechanisms of gene rearrangement in metazoans, additional comparative analyses of non-vertebrate mt genome gene orders within an evolutionary framework are largely wanting.

On the other hand, mt genome arrangement comparisons may be useful for phylogenetic reconstruction [6,7]. For these molecular markers, it is generally assumed that convergence is unlikely because of the great number of potential mt arrangements and the general low rate of rearrangements. Therefore, the presence of rare mtDNA gene orders shared by different taxa can be interpreted as a result of common ancestry. However, structural constraints and the reported existence of hotspots for gene rearrangement (e.g. near the origins of replication in vertebrate mt genomes, [5]) must increase the chances of convergence [5,6]. Both Parsimony [8] and Bayesian [9,10] methods are available to reconstruct phylogenies based on genome arrangement data. However, these methods have several limitations [9], and hitherto have not been extensively applied. Further studies based on larger genome arrangement data sets and more sophisticated methods of inference are expected to confirm the potential of mt genome arrangements as phylogenetic markers, as well as their performance and levels of homoplasy.

**Figure 1:**
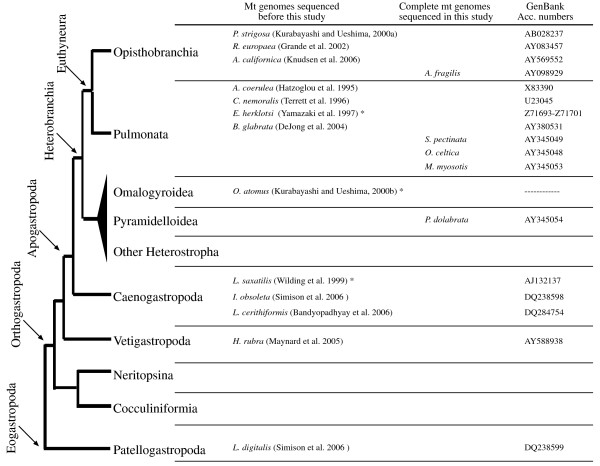
Phylogenetic hypothesis of gastropod relationships based on morphological data as proposed by Ponder and Lindberg [30]. For each lineage, available complete and incomplete (marked with an asterisk) mt genomes are listed together with their corresponding GenBank accession numbers.

Gastropod mollusk mt genomes present high diversity of gene orders [11], and offer a suitable model system to study the rates and mechanisms of mt genome rearrangement, as well as the phylogenetic utility of arrangement comparisons. Thus far, ten complete gastropod mt genomes have been reported including *Albinaria coerulea *[12], *Cepaea nemoralis *[13], *Pupa strigosa *[11], *Roboastra europea *[14], *Biomphalaria glabrata *[15], *Haliotis rubra *[16], *Aplysia californica *[17], *Lottia digitalis *and *Ilyanassa obsoleta *[18], and *Lophiotoma cerithiformis *[19] (Fig. 1). In addition, the incomplete mt genomes of *Euhadra herklotsi *[20], *Littorina saxatilis *[21], and *Omalogyra atomus *[22] have been also described (Fig. 1). These mt genomes are all of relatively reduced size (13–15 Kb) except that of *L. digitalis*, which has two large non-coding regions that increase the total length of the mt genome up to 26 Kb [18].

Any meaningful evolutionary comparison between gastropod mt genome arrangements must rely explicitly on a robust phylogeny of these mollusks. However, phylogenetic relationships among extant groups of gastropods are the subject of a long-standing debate that lasts over a century [23-25]. The current classification of gastropods is a consensus of phylogenetic hypotheses proposed by several authors (for example [26-34]) during the last two decades, and generally includes six major groups: Patellogastropoda, Cocculiniformia, Neritopsina, Vetigastropoda, Caenogastropoda and Heterobranchia (Fig. 1). Although the monophyly of these groups is well supported based both on morphological and molecular evidence, phylogenetic relationships among them are still contentious. Morphological [26,28] but not molecular data [30,34] support Patellogastropoda (Eogastropoda) as the sister group of the remaining gastropods (Orthogastropoda) (Fig. 1). Furthermore, Caenogastropoda and Heterobranchia are considered to be sister taxa (Apogastropoda) based both on morphological and molecular data [28,34,35] (Fig. 1).

Out of the six major gastropod groups, Heterobranchia seems to be the most heterogeneous one. This clade includes the paraphyletic Heterostropha (Omalogyroidea, Pyramidelloidea, and other smaller groups) [26,28], and Euthyneura, a monophyletic group that includes the highly diversified Opisthobranchia and Pulmonata [24] (Fig. 1). The phylogenetic relationships between the paraphyletic heterostrophan lineages and Euthyneura remain unresolved, as well as the reciprocal monophyly of Opisthobranchia and Pulmonata [e.g. [27,28,36,37]]. The absence of a clear morphological distinction between pulmonates (land snails and slugs) and opisthobranchs (sea slugs) has complicated the definition of both groups, with some taxa (eg. Siphonariidae or Onchidiidae) historically included in one or the other group depending on the characters considered [26,38-44]. Moreover, recent mt sequence data analyses tentatively supported the paraphyly of opisthobranchs and the polyphyly of pulmonates [37,45,46].

In this study, we analyze the evolution of mt gene order arrangements within gastropods in order to gain insights on rates and mechanisms of genome rearrangement within the group. We sequenced anew the complete mt genomes of five gastropods species including one representative of Heterostropha (*Pyramidella dolabrata*), one of Opisthobranchia (*Ascobulla fragilis*), and three of Pulmonata (*Siphonaria pectinata*, *Onchidella celtica*, and *Myosotella myosotis*). The newly reported sequences were aligned with all available complete mt genomes of gastropods deposited in GenBank, and subjected to commonly used methods of phylogenetic inference in order to reconstruct a robust phylogeny of gastropods. Genome arrangements of all gastropod mtDNAs were mapped onto the recovered phylogeny in order to determine rearrangement events, and to assess the phylogenetic utility of mt gene order comparisons.

## Results

### Genome structural features

The main structural features of the five gastropod complete mt genomes that were sequenced anew in this study are described in Table 1. Their total length ranges from 13,856 bp (*P. dolabrata*) to 14,745 bp (*A. fragilis*). Their A+T content varies from 55% (*M. myosotis*) to 67% (*A. fragilis*). All of them encode a total of 37 (13 protein coding, 2 rRNA, and 22 tRNA) genes. Of these, 13 genes (*trnQ*, *trnL (uur)*, *atp8*, *trnN*, *atp6*, *trnR*, *trnE*, *rrnS*, *trnM*, *nad3*, *trnS (ucn)*, *trnT*, and *cox3*) are encoded in all the mt genomes by the minus strand.

**Table 1:** Main structural features of the five mt genomes sequenced anew in this study. For each, total size of the mt genome, overall base composition, size and base composition of the potential origin of replication (POR), size of rRNAs, size of intergenic spacers, and size of the protein coding genes (showing start/stop codons within parentheses) are presented. Sizes are expressed as bp.

	*P. dolabrata*	*A. fragilis*	*S. pectinata*	*O. celtica*	*M. myosotis*
total size	13,856	14,745	14,065	14,150	14,246
% A	27.44	30.12	29.76	25.26	23.67
% T	35.97	36.93	37.06	34.06	31.35
% C	16.97	15.13	14.92	18.92	21.35
% G	19.6	17.82	18.26	21.77	23.63
% A+T	63.41	67.05	66,82	59.32	55.02
POR size	42	52	53	43	45
% A+T POR	83.33	86.54	73.58	76.74	60.00
*l-rRNA*	998	1,095	1,022	1,056	1,089
*s-rRNA*	695	738	693	708	712
Intergenic spacers	89	644	229	295	311
*atp6*	640 (ATG/T)	660 (ATA/TAA)	663 (TTG/TAG)	645 (TTG/TAG)	641 (ATA/TA)
*atp8*	163 (ATG/T)	156 (TTG/TAG)	149 (ATG/TA)	147 (ATG/TAA)	151 (ATG/T)
*cob*	1,111 (TTG/T)	1,122 (TTG/TAA)	1,113 (TTG/TAA)	1,122 (ATT/TAA)	1,110 (TTG/TAG)
*cox1*	1,525 (TTG/T)	1,530 (TTG/TAA)	1,528 (TTG/T)	1,527 (TTG/TAG)	1,527 (ATG/TAA)
*cox2*	649 (TTG/T)	684 (TTG/TAA)	665 (TTG/TA)	681 (TTG/TAA)	669 (GTG/TAA)
*cox3*	778 (ATG/T)	778 (ATG/T)	778 (ATG/T)	778 (ATG/T)	778 (ATG/T)
*nad1*	876 (ATT/TAA)	915 (TTG/TAG)	886 (TTG/T)	906 (TTG/TAA)	882 (ATT/TAG)
*nad2*	925 (ATT/T)	942 (ATA/TAA)	935 (TTG/TA)	922 (ATG/T)	948 (ATG/TAG)
*nad3*	351 (TTG/TAA)	354 (TTG/TAG)	350 (ATG/TA)	352 (ATG/T)	335 (ATA/T)
*nad4*	1,332 (TTG/TAA)	1,322 (TTG/TA)	1,328 (TTG/TAA)	1,308 (GTG/TAA)	1,305 (TTG/TAA)
*nad4L*	271 (ATA/T)	283 (ATG/T)	283 (GTG/T)	268 (ATG/T)	291 (TTG/TAA)
*nad5*	1,644 (ATA/TAG)	1,627 (GTG/T)	1,665 (TTG/TAG)	1,643 (GTG/TAG)	1,656 (GTG/TAG)
*nad6*	483 (ATT/TAG)	471 (TTG/TAA)	459 (TTG/TAA)	465 (TTG/TAA)	468 (ATA/TAG)

**Figure 2:**
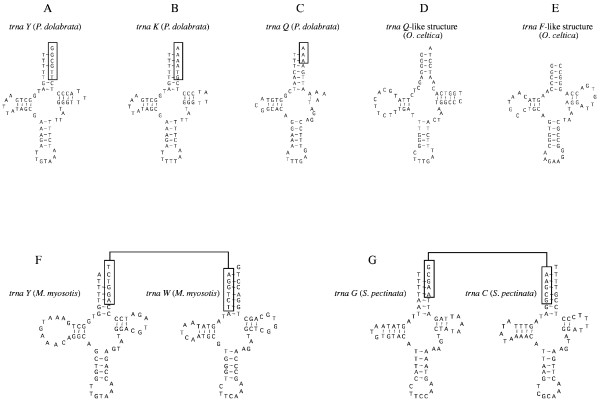
Striking cloverleaf secondary structures of several tRNAs and tRNA-like structures deduced from the complete sequence of the five mitochondrial genomes sequenced in this study. A, B, and C show three proposed cloverleaf secondary structures found in the mt genome of *P. dolabrata*. Boxes in A, B, and C indicate the overlapping nucleotides of these tRNAs with their downstream genes (*cox1*, *trnF*, and *trnC*, respectively). Note that the nucleotide sequences of the *trnY *(A) and *trnK *of *P. dolabrata *(B) are almost identical. A truncated TψC stem is shown in the *trnQ *of *P. dolabrata *(C). D and E indicate potential secondary structures found in the longest non-coding region of the mt genome of *O. celtica*. F and G show several proposed cloverleaf secondary structures found in the mt genome of *M. myosotis *and *S. pectinata *respectively. Boxes in F and G show the overlapping nucleotides between *trnY *and *trnW *in *M. myosotis *(F) and between *trnG *and *trnC *in *S. pectinata *(G).

Overlapping of adjacent genes (even between genes encoded by the same strand) is fairly common in the five mt genomes. In almost all cases the overlap involves tRNA genes, although *cox1 *gene overlaps with *trnK *gene in three mt genomes (*O. celtica*, *M. myosotis*, and *S. pectinata*) and with *trnY *gene in that of *P. dolabrata *(Fig. 2). In addition, *nad4 *gene overlaps with *trnS (agn) *and *trnT *genes in *P. dolabrata*. The total length of intergenic spacers is extremely small for the *P. dolabrata *genome (89 bp), medium for the *S. pectinata*, *O. celtica*, and *M. myosotis *genomes (200–300 bp) and relatively long for the mt genome of *A. fragilis *(644 bp) (Table 1). The longest non-coding region (173 bp) was found in the mt genome of *O. celtica *between *nad6 *and *nad5 *genes. This region was checked for potential secondary structures (stem loops or tRNA-like structures) and repetitive motives. Putative *trnQ*-like and *trnF*-like structures were found (Fig. 2). The potential origin of replication was located in the five mt genomes in a non-coding sequence between *cox3 *and *trnI *genes by comparison with other gastropod genomes. These non-coding sequences (42–53 bp long) have an extremely high A+T content (Table 1).

Initiation and termination codons for the 13 protein coding genes encoded by the five mt genomes are summarized in Table 1. Most protein coding genes start with TTG, although ATG, ATT, ATA and GTG are also common. The ORFs normally end with TAG and TAA stop codons. Incomplete stop codons (T or TA) are common in the two smallest mt genomes (i.e. *P. dolabrata *and *S. pectinata*). Interestingly, *cox3 *gene ends with the incomplete stop codon T in all five mt genomes sequenced in this study.

The 22 tRNAs genes of each of the five mt genomes were identified based on the corresponding anticodons, and their typical cloverleaf secondary structure. All *trnS *genes lack the DHU stem. In some tRNAs genes, the acceptor stem is mispaired (Fig. 2). An extreme reduction in length of the TΨC stem was observed in several tRNAs genes in the mt genome of *P. dolabrata *(*trnF*, *trnQ*, *trnG*, *trnH*, and *trnT*) (Fig. 2). Remarkably, nucleotide sequences of the *trnY *and *trnK *of *P. dolabrata *are almost identical (Fig. 2).

### Phylogeny of gastropods

The deduced amino-acid sequences of 12 mitochondrial protein-coding genes (all except *atp8*) from 15 gastropods and 3 cephalopods were combined into a single data set that produced an alignment of 3,046 positions. Of these, 714 were invariant, and 1,870 were parsimony-informative. Mean character distances among ingroup taxa varied between 0.11 (*I. obsoleta *and *L. cerithiformis*) and 0.60 (*L. digitalis *and *C. nemoralis*). The average mean character distance was 0.37 (± 0.07) among Heterobranchia lineages, 0.46 (± 0.02) between Heterobranchia and Caenogastropoda, 0.53 between *L. digitalis *and Caenogastropoda, and 0.57 (± 0.01) between *L. digitalis *and Heterobranchia.

**Figure 3:**
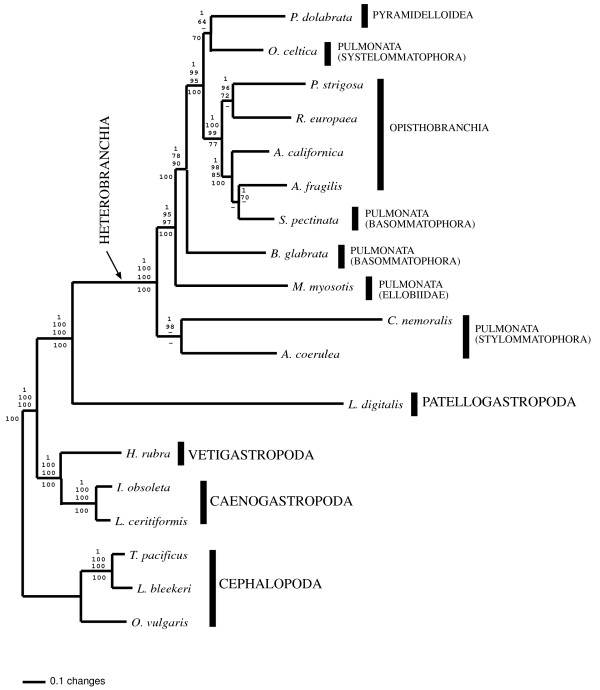
Phylogenetic relationships among Gastropoda. The 50%-majority rule consensus of post-burn-in sampled trees from the Bayesian inference analysis based on a data set including the deduced amino acid sequences of all the protein coding genes (except *atp8*) of all gastropod complete mt genomes sequenced so far is shown. Branch lengths are mean estimates. Three species of cephalopods were used as outgroup taxa. Numbers in the nodes are from top to bottom: BI BPP, and ML, MP, and ME BP. Dashes indicate BP below 70%.

The reconstructed Bayesian 50% majority rule consensus tree (-lnL= 68443.87) based on the concatenated data set is shown in figure 3. All nodes received maximal BPP support. MP (12,933 steps; CI = 0.67), ME (score = 3.10), and ML (-lnL = 68,436.70) arrived at identical topology. All nodes received high BP support except the sister group relationships of *P. dolabrata *+ *O. celtica *and *P. strigosa *+ *R. europaea *in the MP and ME analyses, respectively (Fig. 3). In addition, neither MP nor ME analyses supported the clade *A. fragilis *+ *S. pectinata*. BI based on an amino acid data set considering each mt gene as a different partition, and using a partitioned mixed model of the combined data recovered an almost identical tree (-lnL= 69881.74). The only differences to the tree shown in Figure 3 were that *S. pectinata *was recovered as sister group of *A. californica *to the exclusion of *A. fragilis *with low BPP support (91%), and that the sister group relationship of *P. dolabrata *+ *O. celtica *was not supported. All the other nodes received maximal BPP support (not shown). An identical topology to that shown in Figure 3 was recovered when the polyplacophoran *Katharina tunicata *was used as outgroup (not shown).

Four main lineages were identified within gastropods: Caenogastropoda (including *I. obsoleta *and *L. ceritiformis*), Vetigastropoda (*H. rubra*), Patellogastropoda (*L. digitalis*) and Heterobranchia (all remaining analyzed species). According to the recovered tree, Caenogastropoda and Vetigastropoda are sister group taxa, as well as Patellogastropoda and Heterobranchia (Fig. 3). Within Heterobranchia, neither Euthyneura nor Pulmonata nor Opistobranchia were recovered as monophyletic groups (Fig. 3). The monophyly of Euthyneura was strongly rejected because the heterostrophan *P. dolabrata *was recovered deep within Pulmonata + Opistobranchia (Fig 3). The recovered relative positions of Stylommatophora, Ellobiidae, Basommatophora (non-monophyletic), and Systelommatophora render Pulmonata polyphyletic whereas Opisthobranchia is recovered paraphyletic due to the inclusion within this group of the basommatophoran pulmonate *S. pectinata *(Fig. 3).

The AU, SH and KH tests (Table 2) were performed to evaluate whether alternative morphology-based hypotheses could be rejected based on the analyzed mt data set. The AU and KH test values for the sister group relationship of Eogastropoda and Orthogastropoda, and the monophyly of Apogastropoda, Euthyneura, Pulmonata, and Basommatophora were = 0.01 in all cases. For the SH test, all values for alternative hypotheses were = 0.01 except for the sister group relationship of Eogastropoda and Orthogastropoda, and the monophyly of Apogastropoda. Overall, the performed tests showed that alternative morphological hypotheses could be statistically rejected based on our data set.

**Table 2:** Statistical tests of alternative phylogenetic hypotheses

Topology*		Loglikelihood	AU test	KH test	SH test
Unconstrained	((out),(((Iob, Lce),Hru),(Ldi,((Cne,Aca),(Mmy,(Bgl,((Pdo,Oce),((Pst,Reu),(Acal,(Afr,Spe))))))))))	-67745.05	0.99	0.99	1.00
Orthogastropoda	((out),(Ldi,((Hru,(Iob,Lce)),((Cne,Aca),(Mmy,(Bgl,((Oce,Pdo),((Pst,Reu),(Acal,(Afr,Spe))))))))))	-67771.22	< 0.01	0.01	0.50
Apogastropoda	((out),(Hru,(Ldi,((Iob,Lce),((Cne,Aca),(Mmy,(Bgl,((Oce,Pdo),((Pst,Reu),(Acal,(Spe,Afr)))))))))))	-67827.85	< 0.01	< 0.01	0.07
Euthyneura	((out),(((Iob,Lce),Hru),(Ldi,(Pdo,((Cne,Aca),(Mmy,(Bgl,(Oce,((Pst,Reu),(Acal,(Afr,Spe)))))))))))	-67853.51	< 0.01	< 0.01	0.01
Basommatophora	((out),((Hru,(Iob,Lce)),(Ldi,((Cne,Aca),(Mmy,((Bgl,Spe),((Oce,Pdo),((Pst,Reu),(Acal,Afr)))))))))	-68018.04	< 0.01	< 0.01	< 0.01
Pulmonata	((out),((Hru,(Iob,Lce)),(Ldi,(((Bgl,Spe),((Oce,(Cne,Aca)),Mmy)),(Pdo,((Pst,Reu),(Acal,Afr)))))))	-68106.99	< 0.01	< 0.01	< 0.01
Traditional	((out),(Ldi,(Hru,((Iob,Lce),(Pdo,(((Bgl,Spe),((Oce,(Cne,Aca)),Mmy)),((Pst,Reu),(Acal,Afr))))))))	-68160.24	< 0.01	< 0.01	< 0.01

* out = outgroup; Iob = *Ilyanassa obsoleta *; Lce = *Lophiotoma cerithiformis *; Hru = *Haliotis rubra *; Ldi = *Lottia digitalis *; Cne = *Cepaea nemoralis *; Aca = *Albinaria coerulea; *Mmy = *Myosotella myosotis *; Bgl = *Biomphalaria glabrata *; Pdo = *Pyramidella dolabrata; *Oce = *Onchidella celtica *; Pst = *Pupa strigosa *; Reu = *Roboastra europaea; *Acal = *Aplysia californica *; Afr = *Ascobulla fragilis *; Spe = *Siphonaria pectinata*

### Evolution of gastropod mt genome arrangements

**Figure 4:**
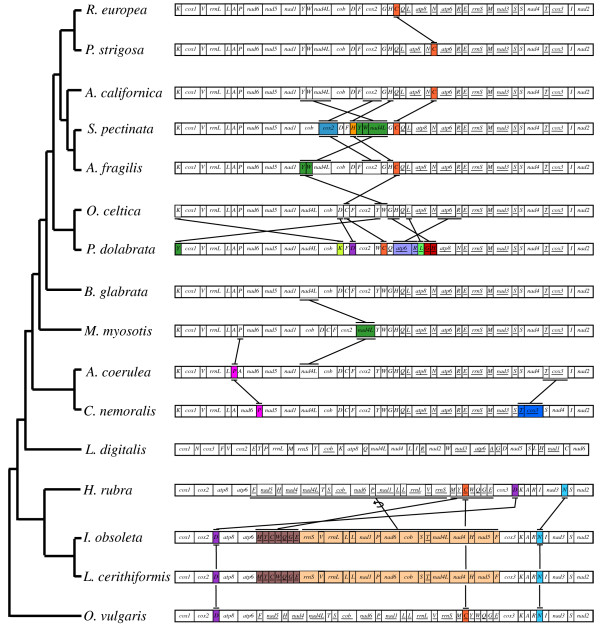
Hypothesized mitochondrial gene rearrangements during Gastropoda evolution based on observed gene orders and the recovered BI phylogenetic hypothesis. Inversion (indicated by the arrow) and transpositions of protein coding, tRNAs and rRNA genes are depicted among the different taxa (except between *H. rubra*-*L. digitalis *and *H. rubra*- Heterobranchia due to the high number of changes). Genes encoded by the minor strand are underlined. Genes located in apomorphic arrangements are colored.

Each of the five newly determined gastropod mt genomes exhibits a different gene order (Fig. 4). Moreover, among all reported gastropod mt genomes only two sets of species (*I. obsoleta *+ *L. cerithiformis*, and *A. californiaca *+ *P. strigosa*) have the same gene order (Fig. 4). Despite the observed arrangement diversity, several general patterns may be inferred. The gene order of the *L. digitalis *mt genome is the most divergent among all gastropod mtDNAs sequenced thus far. The vetigastropoda *H. rubra *mt genome has the same gene order of the cephalopod *O. vulgaris *mt genome except for the relative position of three tRNA (*trnC, trnD *and *trnN*) genes (Fig. 4). The identical gene order exhibit by the two caenogastropod mt genomes only differs from that of *O. vulgaris *in one translocation and one inversion (Fig. 4). Despite several autapomorphies, all heterobranch mt genomes analyzed in this study share a general conserved gene order, which substantially differs from that of other gastropods (Fig. 4). *S. pectinata *and *P. dolabrata *show several autapomorphies that involve changes not only in the relative position of tRNAs but also of some protein coding genes (*cox2*, *nad4L*, and *atp6*) (Fig. 4). *M. myosotis *and *C. nemoralis *have autapomorphic relative positions for *nad4L *and *cox3 *genes, respectively (Fig. 4). Changes in the remaining analyzed heterobranch mt genomes affect the arrangement of *trnW*, *trnY*, *trnC*, *trnP*, and *trnT *genes (Fig. 4).

Genome arrangements were mapped onto the phylogenetic hypothesis presented in figure 3 in order to gain insights on the patterns, rates, and mechanisms of gastropod mt genome rearrangements. The evolutionary comparative analyses showed that most gene rearrangements occurred in the lineages leading to Patellogastropoda and Heterobranchia, whereas Vetigastropoda and Caenogastropoda mostly retain the ancestral gene arrangement (as represented in the analysis by the cephalopod mt genome arrangement). Within Heterobranchia, rates of rearrangement seem to have significantly slowed down, and accelerated again in the lineages leading to *P. dolabrata *and *S. pectinata*. Most inferred rearrangements involve tRNA genes rather than protein coding genes. Furthermore, changes in gene order are normally related with translocations, most moving to proximal regions but also some to more distant regions. Among all inferred rearrangements, only one instance of gene inversion between *H. rubra *and the ancestor of the Caenogastropoda was found. In most cases, rearrangements involve one or few genes. However, the described inversion involved a genome fragment with 16 genes.

Attempts to recover phylogenetic relationships among gastropods based on genome arrangement information and using parsimony- or Bayesian-based methods of phylogenetic inference rendered highly unresolved trees (the only sister-group relationships that were confidently recovered were *A. californica *+ *P. strigosa *and *I. obsoleta *+ *L. cerithiformis*, which each have identical gene order) (data not shown).

## Discussion

### Evolutionary trends in the structural features of gastropod mt genomes

The mt genomes of the gastropods sequenced so far contain the 37 genes described for the majority of mt genomes within Metazoa [47]. This is not the case for other groups of mollusks like bivalves, which lack some genes (e.g. *atp8*, [48], but see [49]) or both bivalves and cephalopods, which show duplicated genes (e.g. *rrnS*, *cox1*, *cox3*, *atp6*, and *atp8*) [50-52]. This observation suggests that changes in gene content and number might be exclusive features characterizing bivalves and cephalopods (unless new data from other groups of mollusks points otherwise), and that gastropods maintain the ancestral number of genes with no translocations of genes to the nucleus and/or any signal of duplication events (but see below for striking exceptions to this general rule).

The compact organization, gene order, and molecular features of the five heterobranch mt genomes sequenced anew in this study fit well within the general description of the gastropod mt genomes that have been sequenced so far [11-20]. The small lengths of the genes and intergenic spacers, as well as the high number of overlaps between adjacent genes render gastropod mt genomes amongst the smallest in size within Metazoa. The only exception to this general trend is the relatively larger size of the mt genome of the patellogastropodan *L. digitalis*, which is due to the presence of several non-coding tandem repeat units.

Several hypotheses have been proposed to explain overlapping of adjacent mt genes that are transcribed from the same strand, including the existence of multiple promoters, differential cleavage to generate diverse RNAs or post-transcriptional editing of the RNA [48]. All overlapping events that can be observed in the five mt genomes described in this study are between adjacent tRNA genes or between adjacent tRNA and protein coding genes and could be explained by tRNA post-transcriptional mechanisms as described by Yokobori and Pääbo [53]. Observed overlaps in the five gastropod mt genomes are restricted to the acceptor stem, where the 5' end of the tRNAs overlaps with the downstream gene. The nucleotides that overlap seem to be part of the downstream gene while the complementary nucleotides needed in the acceptor stem of the tRNA genes could be added by polyadenylation [53]. The presence of truncated TΨC arms in many tRNA genes in *P. dolabrata *could be the result of the extreme reduction in length of the genes in the mt genome of this species.

Two tRNA-like structures (*Q*-like and *F*-like) were found in the mt genome of *O. celtica*, in a non-coding region between the protein coding genes *nad6 *and *nad5*. These tRNA-like structures could result from duplication events in the mt genome of *O. celtica *and may have been able to remain as pseudogenes because they were located in a non-coding region. It has been suggested that mt genes are expressed as a polycistron, and that tRNA-like structures might be related to the processing of the primary transcripts liberating the flanking gene specific mRNAs [20]. Due to the relevance of this hypothetical function, one would expect to find these structures in high frequencies in mt genomes. However, many protein coding genes in the mt genomes of gastropods abut directly (without tRNA genes or tRNA-like structures between them) and only in very few cases some secondary structures have been described [20]. Therefore, the genome organization data available so far do not support the above-mentioned hypothesis as a general mechanism for processing mRNAs in mt genomes of gastropods, although further studies based on the transcription and processing of mt genes in this group are needed.

There is no general pattern in the use of initiation and termination codons in the 13 protein coding genes of the five mt genomes analyzed in this study. Although a strand-specific pattern of termination codon usage has been described for the gastropod *H. rubra *[16], this does not seem to be a general feature for gastropod mt genomes. The use of incomplete termination codons (T, TA) is especially high in the two mt genomes with smallest sizes (i.e. *P. dolabrata *and *S. pectinata*). Post-transcriptional poly-adenylation could generate the complete termination codon [15,20]. Our results suggest that *cox3 *might be a very conserved gene both in length and in codon usage within Gastropoda.

### New insights into the phylogeny of Gastropoda

The reconstructed gastropod phylogeny based on 12 mt protein-coding gene sequence data supports four natural groups within Gastropoda: Vetigastropoda, Caenogastropoda, Patellogastropoda, and Heterobranchia. The systematic validity of these groups is in full agreement with most recent phylogenetic analyses based on morphological and molecular data [26,28,34]. Morphological analyses also distinguish two additional lineages within gastropods including Neritopsina [28] and the enigmatic Cocculiniformia [26]. Mt genomes of representatives of these two groups need to be sequenced in the future to test their phylogenetic position within gastropods.

Although many efforts have been made in the last years to resolve phylogenetic relationships among the main lineages within Gastropoda, many questions remain open. One of the most controversial issues refers to the position of Patellogastropoda. Morphological studies consistently place patellogastropods as the sister taxon to the rest of gastropods [25,26,28,42,54]. However, Colgan et al. 2003 [34] proposed a reevaluation of the morphological characters defining Patellogastropoda and its relationships with other gastropods. The morphological dataset was updated with new data about buccal cartilages and the fine structure of the cephalic tentacles, and the validity of previous polarization of some characters like the absence of the hypobranchial gland or the flexoglosste condition of the radula was questioned [34]. Morevover, molecular studies produced conflicting results, recovering unstable positions for Patellogastropoda [30,34]. In our phylogenetic analyses, *L. digitalis *is recovered as the sister group to Heterobranchia with statistical support when trees are rooted with both cephalopods and polyplacophorans (a sister group relationship between Eogastropoda and Orthogastropoda was rejected by the AU and KH (but not SH) tests; Table 2). The mt genome of *L. digitalis *exhibits a unique mt gene order, and in the phylogenetic analyses, this taxon has a long branch compared to that of other gastropods. A number of studies have suggested that nucleotide substitution and gene rearrangement rates may be correlated in mitochondrial genomes (see [55] and references therein). This may be the case in *L. digitalis*, and long-branch attraction phenomena could likely be biasing the inference of the phylogenetic position of this species, particularly taking into account that any outgroup taxa that could be included in the phylogenetic analyses are all distantly related to gastropods. The complete mt genomes of more representatives of Patellogastropoda need to be analyzed in order to resolve the phylogenetic position of Patellogastropoda and to discern among competing hypotheses.

Previous morphological and molecular studies based on nuclear markers recovered Caenogastropoda and Heterobranchia as sister taxa, conforming the Apogastropoda [26,28,31,35,56]. However, all the analyses performed in this study support a strikingly close relationship between Vetigastropoda and Caenogastropoda. The AU and KH (but not SH) tests consistently rejected the validity of Apogastropoda (Table 2). Morphological synapomorphies described for Apogastropoda should be revisited taking into account our results, and perhaps new synapomorphies for Vetigastropoda and Caenogastropoda may be found.

The monophyly of Heterobranchia is well supported by several morphological synapomorphies like the presence of pigmented mantle organs, longitudinal rows of cilia in the mantle cavity, a chalaze in the egg masses, heterostrophy, and simultaneous hermaphroditism [57]. Phylogenetic analyses based on mt genome sequence data support the monophyly of heterobranchs (Fig. 3). However, the monophyly and phylogenetic relationships between the different groups included within Heterobranchia (i.e. Heterostropha and Euthyneura (Pulmonata and Opisthobranchia)) have been the subject of controversy for many years [25,26,58-60]. Heterostropha was defined as a paraphyletic group including species with typically ancestral and derived characters mixed together in the same forms [26,28,36]. The unique representative of Heterostropha included in this study (*P. dolabrata*) belongs to Pyramidelloidea, a group of gastropods that has been excluded of Heterobranchia by some authors [23,25,61], placed as a basal heterobranch with respect to Euthyneura [58], or included in Opisthobranchia by others [39,62-65] depending on the characters considered. All our analyses recover *P. dolabrata *as closely related to systelommatophoran pulmonates and opisthobranchs, and confirm our previous studies [37].

Pulmonata includes marine, freshwater and terrestrial gastropods with very different body plans. The monophyly of Pulmonata has been accepted by many authors based on some morphological characters like the streptoneuran inervation of the cephalic tentacles, and the lack of rhinophoric nerve (present in opisthobranchs and pyramidellids). However, the essential, traditionally accepted morphological synapomorphy of Pulmonata is the presence of a special neurosecretory system comprising procerebrum and cerebral gland [26,66-68]. The procerebrum is formed of small and large neuronal cells, and because it links the peripheral tentacular structures with the central nervous system, an olfactory function has been assumed. The cerebral gland is a neuronal structure associated with the cerebral ganglia. New molecular data reject Pulmonata as a natural group based on both nuclear and mitochondrial data [32,37,45]. In this study, we have included representatives of all major lineages within pulmonates (Systelommatophora, Basommatophora, Ellobiidae, and Stylommatophora). All these lineages independently reject the definition of pulmonates as a natural group in all the performed analyses (Fig. 3; Table 2). Stylommatophora (land snails) is a monophyletic group, in agreement with previous morphological studies [69], and it is recovered as the sister group to all other heterobranchs studied (Fig. 3). Our results provide new insights into land colonization by heterobranch gastropods. The transition to a land lifestyle was accompanied by a variety of refined morphological and physiological modifications. As a result, land snails and slugs constitute a well-defined group of pulmonates with several morphological synapomorphies in the cephalic tentacles, kidney, and central nervous system, as well as in several aspects of their ontogeny. Previous phylogenetic hypotheses had suggested that the transition to land was a rather derived event in the history of pulmonates. Our molecular phylogeny instead supports a different scenario in which gastropod land colonization, and subsequent radiation was an early and significant event in the evolution of Heterobranchia.

The marine Basommatophora *S. pectinata *is more closely related to opisthobranchs than to the freshwater basommatophoran *B. glabrata *or to any other group of pulmonates considered in this study (Fig. 3). The phylogenetic affinities of *S. pectinata *have been under debate for many years (see discussion in [37]). All new data and analyses presented here support *S. pectinata *as an opisthobranch. To test the monophyly of freshwater basommatophorans, mt genome sequence data of more representatives of this group should be included in future analyses.

The monophyly of pulmonates is also rejected by the location of a representative of Systelommatophora (*O. celtica*) as an independent linage more closely related to *P. dolabrata *and to opisthobranchs than to any other Pulmonate (Fig. 3), which corroborates previous morphological hypotheses [58,70].

The robust results described in this study provide new insights on the systematics of gastropods. The majority of our evolutionary hypotheses are in agreement with traditional morphological hypotheses although there are some cases of strong discrepancy (i.e. the phylogenetic position of Patellogastopoda, the sister group relationship between Vetigastropoda and Caenogastropoda, and the polyphyly of Pulmonata). Among these results, the polyphyly of pulmonates is perhaps the most remarkable, and warns against only relying on one or few morphological characters (even if they seem to be free of convergent evolution) to define deep phylogenetic relationships. In any case, the results here presented should be interpreted as a working phylogenetic hypothesis, which needs to be further confirmed with a larger taxon sampling of the studied groups, and the addition to the phylogenetic analyses of new taxa representing not previously included major lineages of gastropods.

### Mitochondrial genome rearrangements during the evolution of Gastropoda

Gene order rearrangements in mt genomes are relatively rare, and if shared derived by two taxa can be considered molecular synapomorphies and may provide useful data for phylogenetic reconstruction. In this study, we have mapped gene orders of gastropod mt genomes onto the gastropod phylogeny and tentatively reconstructed the evolutionary history of mt gene order rearrangements in gastropods. The Vetigastropoda *H. rubra *and the cephalopod *O. vulgaris *have the same gene order (with only three changes in *trnC, trnD *and *trnN*) suggesting that *H. rubra *may retain the ancestral mt gene order of Gastropoda. The relative placement of *trnD *and *trnN *in *H. rubra *might constitute autapomorphies in this species since these two tRNAs show the same location in Caenogastropoda and the cephalopod *O. vulgaris*.

Considering all the data available so far, three major rearrangements have taken place in the evolutionary history of gastropods: one in the ancestor of Caenogastropoda, another in the ancestor of Patellogastropoda, and another one in the ancestor of Heterobranchia.

The two complete mt genomes of caenogastropods sequenced so far and the incomplete mt genome of the caenogastropod *L. saxatilis *have identical gene arrangements (data not shown). Two rearrangements (one inversion and one translocation) separate the hypothetical ancestral state of gastropods from the gene order found in Caenogastropoda (Fig. 4). Long inversion events rendering new gene order configurations have been already described for other closely related groups [71].

The mt gene order of *L. digitalis *is very distinctive compared to those other mollusk mt genomes sequenced so far. The remarkable number of gene rearrangement events that need to be invoked to explain the mt gene order of *L. digitalis *requires considering special mechanisms such as intramolecular recombination facilitated by tandem repeat sequences, as described for other metazoans [72]. Sequencing of other patellogastropodan mt genomes should determine whether the striking gene order of *L. digitalis *is unique to this species or a more general feature of Patellogastropoda.

The mt gene order of the heterobranchs sequenced so far, including those of the incomplete mt genomes of the Stylommatophoran *E. herklotsi *and the Heterostrophan *O. atomus *(data not shown), has only few gene boundaries in common with the hypothetical ancestral mt gene order of gastropods. Considering all the data available so far, it is not possible to determine the precise mechanism responsible for rearrangements in this transition (tandem duplication-random loss [73], inversion [71], transposition [74], and/or intramolecular recombination [72]). The compact organization of the mt genomes of Heterobranchia (with very few and short non coding sequences) suggests strong selection against maintaining remnants of duplication events. However, the *trnF*-like structure described in this study, might represent one remnant of a putative duplication event, and would be in support of tandem duplication and random loss as a mechanism acting in heterobranch mt genome rearrangements.

The new data presented here suggest that the gene order among heterobranchs is not as well conserved as previously thought [11,14,17,20,22]. Most part of these mt genomes shows a rather conserved gene order, being gene rearrangements concentrated between *nad1 *and *trnE *genes (Fig. 4). Several gene order autapomorphies (not only in tRNAs but also in protein coding genes) can be detected in heterobranchs, especially in *S. pectinata *and *P. dolabrata*.

Two additional aspects about gene order in heterobranchs can be highlighted. First, the gene order *trnY*-*trnW*-*nad4L *supports the close relationship between opisthobranchs and *S. pectinata*. Second, *trnY *bounds with *cox1 *gene in the mt genome of *P. dolabrata *(instead of *trnK *gene as in all other heterobranchs). However, the sequences of the *trnY *and *K *genes in *P. dolabrata *are nearly identical (Fig. 2), suggesting that two mutation events might have occurred in the anticodon triplet producing changes in the identity of these two tRNAs. This event, called tRNA recruitment, has also been previously described in other taxa [75].

The phylogenetic inferences based on genome arrangement data rendered inconclusive results. Despite the relatively high number of different gene orders found in gastropod mt genomes, few of them are shared and derived, and thus both parsimony and Bayesian inference arrived at rather unresolved trees. Our results point out that the comparative study of gene arrangement in gastropods may provide valuable phylogenetic information, but that current methods of phylogenetic inference based on gene arrangements still need further development.

## Conclusion

According to our results, the validity of Apogastropoda should be questioned, Pyramidelloidea should be included within Euthyneura, *S. pectinata *should be recognized as a member of Opisthobranchia, and Pulmonata should not be considered a natural group. Our results stress the need of a thorough re-evaluation of the morphological characters that have been used to analyze relationships within Gastropoda, and in particular those that supported the monophyly of pulmonates. Although the number of complete mt genomes is increasing rapidly in the last years, still more genomic data is needed to further understand gastropod systematics. The phylogenetic affinities of Neritopsina and Cocculiniformia with respect to other gastropods, the position of Patellogastropoda among gastropods, and the delimitation of Heterostropha and Euthyneura are still open questions that could be tackle in the future with the approach proposed in this study. The recovered phylogeny based on complete mt genome data provides a first instance of robust phylogenetic framework for comparative studies in gastropods, and will allow a better understanding of evolutionary trends within this group. In particular, it seems to be quite useful and promising for determining the rates and mechanisms of gene rearrangement in gastropod mt genomes.

## Methods

### Taxon sampling

Each of the five gastropod complete mt genomes sequenced anew in this study was obtained from a single specimen. *A. fragilis *was collected in Murcia (southeastern Iberian Peninsula) and preserved frozen at -20°C. *S. pectinata *and *O. celtica *were sampled in Ceuta (northern Africa) and preserved in EtOH 100%. *P. dolabrata *was collected in Annobon Island (western Africa) and preserved in EtOH 100%. *M. myosotis *was collected in Vigo (northwestern Iberian Peninsula) and preserved frozen at -20°C. All specimens were sampled between 2000 and 2002.

### DNA extraction, PCR amplification, cloning and sequencing

Total cellular DNA was purified following a standard phenol/chloroform extraction [76]. Universal primers were used to amplify by polymerase chain reaction (PCR) fragments of the mitochondrial *cox1 *(LCO-1490 and HCO-2198, [77]), *rrnL *(16Sar-L and 16Sbr-H, [78]), and *rrnS *(H1478 and L1091, [79]) genes. Standard PCR reactions containing 67 mM Tris-HCl, pH 8.3, 1.5 mM MgCl_2_, 0.4 mM of each dNTP, 2.5 μM of each primer, template DNA (10–100 ng), and Taq DNA polymerase (1 unit, Biotools) in a final volume of 25 μl were subjected to 30 cycles of denaturing at 94°C for 60 s, annealing at 42°C for 60 s, and extending at 72°C for 90 s. The PCR amplified fragments were sequenced with the BigDye Deoxy Terminator cycle-sequencing kit (Perkin Elmer Biosystems) in an automated DNA sequencer (ABI PRISM 3100) using the PCR primers, and following manufacturer's instructions.

The sequences of these fragments were used to design three sets of specific primers for each species that amplified, by long PCR, three fragments that covered the remaining mt genome. Long PCRs containing 60 mM Tris-SO_4 _(pH 9.1), 18 mM (NH_4_)_2 _SO_4_, 1–2 mM MgSO_4_, 0.2 mM of each dNTP, 0.4 μM of each primer, and Takara enzyme (1 unit; Life Technologies) in a final volume of 50 μl were subjected to 40 cycles of denaturing at 94°C for 30 s, annealing at 52°C for 30 s, and extending at 68°C for 7 min. Long PCR products in some cases and total cellular DNA extractions in others were used as DNA templates to amplify by standard PCR reactions (see conditions above) overlapping fragments that covered the complete mt genomes. These overlapping PCRs were performed using degenerated primers (designed based on published mt genome sequences of gastropods) and/or specific walking primers for each species. The sequences of all these primers are available from the authors upon request. PCR products were cloned into the pGEM-T vector (Promega), and sequenced using M13 universal primers in an automated sequencer (see above).

### Molecular and Phylogenetic analyses

Gene annotation was performed using BLAST [80] comparisons against published gastropod mtDNAs. Sequence data were handled with MacClade version 4.05 OSX [81] and PAUP* version 4.0b10 [82]. Protein coding genes were recognized by inferring open reading frames (ORFs), and by delimiting start and stop codons. Cloverleaf secondary structures of all tRNA genes were reconstructed by hand upon localization of the specific anticodons. The sequences of the complete mt genomes reported in this paper have been deposited at the EMBL/GenBank data libraries under accession numbers AY345054 (*P. dolabrata*), AY098929 (*A. fragilis*), AY345049 (*S. pectinata*), AY345048 (*O. celtica*), and AY345053 (*M. myosotis*).

Phylogenetic analyses included the five newly determined mtDNA sequences, as well as all gastropod complete mt genome sequences available in GenBank. In addition, the corresponding sequences of the cephalopod species *Octopus vulgaris*, *Todarodes pacificus *[52], and *Loligo bleekeri *[83], and the polyplacophoran *Katharina tunicata *[84] were used as outgroups. The deduced amino-acid sequences of each mt protein-coding gene (except *atp8*) were aligned independently using Clustal X version 1.62b [85] followed by refinement by eye in an effort to maximize positional homology. Ambiguous alignments and gaps were discarded from further phylogenetic analyses. The aligned amino acid sequences were concatenated into a single data set, which was subjected to maximum-parsimony (MP), minimum evolution (ME), maximum likelihood (ML), and Bayesian inference (BI) methods of phylogenetic reconstruction. MP analyses were performed with PAUP* using heuristic searches (TBR branch swapping; MulTrees option in effect) with 10 random additions of taxa. ME analyses [86] were carried out with PAUP* using mean character distances. Robustness of the resulting MP and ME trees was evaluated with non-parametric bootstrap proportions (BPs, [87]) as implemented in PAUP* with 1,000 pseudoreplicates. ProtTest version 1.3 [88] was used to estimate the evolutionary model that best fit the amino-acid data set. The Akaike information criterion (AIC) implemented in ProtTest selected the WAG+I+G [89] evolutionary model. ML analyses using the WAG+I+G were performed with PHYML version [90], and robustness of the resulting ML tree was evaluated by bootstrapping with 500 pseudoreplicates. BI analyses were performed with MrBayes 3.12 [91] by simulating a Metropolis-coupled Markov chain Monte Carlo (MCMCMC) with four simultaneous chains, each of 10^6 ^generations (sampled every 100 generations) under the WAG+I+G model. Trees sampled before the cold chain reached stationarity (as judged by plots of ML scores) were discarded as "burn-in". Runs were repeated twice. Robustness of the resulting BI tree was evaluated using Bayesian posterior probabilities (BPPs). In addition, BI was also applied to a data set that included the aligned amino acid sequence of each gene as independent partitions. The AIC implemented in ProtTest was used to select the substitution models for each gene: *atp6 *(RtREV+G+F), *cox1 *(WAG+G+F), *cox2 *(RtREV+I+G+F)*, cox3 *(cpREV+G+F)*, cob *(cpREV+I+G+F)*, nad1 *(RtREV+G+F)*, nad2 *(WAG+I+G+F)*, nad3 *(RtREV+G+F)*, nad4 *(WAG+I+G+F)*, nad4L *(MtREV+G+F)*, nad5 *(RtREV+I+G+F)*, nad6 *(Dayhoff+G+F). This data set was subjected to the same searching parameters for BI described above plus the "set partition" and "unlink" options.

Alternative phylogenetic hypotheses were tested using the approximately unbiased (AU), Shimodaira- Hasegawa (SH), and Kishino-Hasegawa (KH) tests [92] as implemented in CONSEL version 0.1 [93] using default settings. The alternative hypotheses tested were *a priori *morphology-based hypotheses.

Phylogenetic relationships among gastropods were also reconstructed based on genome arrangement data using parsimony and Bayesian inferences with the GRAPPA [8] and Badger [94] programs.

## Abbreviations

BI: Bayesian inference; ME: minimum evolution; ML: maximum likelihood; MP: maximum-parsimony; mt: mitochondrial. BPP: Bayesian posterior probabilities; BP: Bootstrap proportions;

## Authors' contributions

JT collected some specimens for this study. CG and RZ gathered the sequences, and performed phylogenetic analyses. All authors wrote the manuscript, read and approved the final version of the manuscript.
